# Paralysis of the Nerve Radialis

**Published:** 1895-08

**Authors:** Otto Noack

**Affiliations:** Reading, Pa.


### REPORTS OF CASES.

### Paralysis of the Nerve Radialis.[1]

BY OTTO NOACK, V.S.,
READING, PA.

The nerve trunks are divided into nerves of sensation and motor nerves. Likewise we have to divide the affections of the nerve trunks into two groups. In one the chief symptoms are loss of muscular power, in the other loss of cutaneous sensibility; these are generally called peripheral paralyses to distinguish them from similar affections due to diseases of the brain or spinal cord. To this class belongs the paralysis of the nerve radialis.

*Causes.* There are many different causes of the peripheral paralysis. It may be due to lesions or other affections of the nerve trunk, or its neighborhood, and the inflammation that is caused thereby. Sometimes it is the result of severe accidents, but similar symptoms frequently follow simple pressure of the nerves or an injury so slight that its occurrence may be overlooked. A similar effect may also be produced by lying on some object. It is an impossibility also in this case to say what the cause is; no lesion or injury can be seen.

By the driver's report I learned that the horse commenced to limp when he took him out of the stable in the morning, and after driving about one mile he could not get him further, because the horse could use only three legs.

*Symptoms.* By ocular inspection no one would suspect that any lameness was there; by standing the horse's weight was equally divided on all legs; only by closer examination one could see the olecranon muscles seemed to be weak and without power, and by testing could feel the loss of strength. Having the horse walked I find him raising the affected leg, and right away losing all control of it; the leg only could be brought in its normal place by assistance. It was easy to diagnose that it was a paralysis of a motor nerve, as the horse had great pain by the slightest touch. These symptoms were so characteristic that I could state at once a paralysis of the nerve radialis.

[1] Reported to the Schuylkill Valley Veterinary Medical Association.

*Prognosis.* In all cases of this kind of paralysis we can say the prognosis is favorable. I saw in my practice about five of these cases, and every one came all right by proper treatment. And so I gave the owner a favorable prognosis with the statement that in about three weeks' time the horse could be used.

*Treatment.* Of many methods of treatment electricity would be recommended and would perhaps be successful, but I must say that electricity cannot well be applied to horses, because the animals get so frightened and so wild by the first application that they begin to kick on seeing the electrical apparatus. I consider the best method of treating these cases is to apply massage of the muscles, and to prevent the turning in of lower joint by putting on a shoe with a very long toe, that the horse is compelled to avoid the weakness in the ankle and to bring the full weight on the hoof. Then you can apply some kind of irritating salves or blistering. Very often by using hot vinegar applications it gives good results. I have cured with this treatment a case of paralysis of the facial nerve—also ingestions of sulphate of veratria will give the same effect. In this case I used at first the application of 6 per cent. solution of spirits of mustard, and afterward, when the swelling was away, due to the mustard, I applied massage to the muscles and a shoe with long toe, and every day exercising the horse for about one-half to one hour. After three weeks' treatment the horse could be put in service again. And this treatment never failed in all the cases of paralysis of the radial nerve in my practice.

Gentlemen, I selected as my subject this case that I had to treat lately, thinking it one you will very seldom see, and I think it is of great interest. The affected horse belonged to the firm of Winter & Goetz, of Reading, and was a bay gelding 15.3 high, six years of age, white stockings on the hind legs.

